# Improved ^223^Ra Therapy with Combination Epithelial Sodium Channel Blockade

**DOI:** 10.2967/jnumed.121.261977

**Published:** 2021-12

**Authors:** Diane S. Abou, Amanda Fears, Lucy Summer, Mark Longtine, Nadia Benabdallah, Ryan C. Riddle, David Ulmert, Jeff M. Michalski, Richard L. Wahl, Denise Chesner, Michele Doucet, Nicholas C. Zachos, Brian W. Simons, Daniel L.J. Thorek

**Affiliations:** 1 Mallinckrodt Institute of Radiology, School of Medicine, Washington University in St. Louis, St. Louis, Missouri;; 2 Program in Quantitative Molecular Therapeutics, School of Medicine, Washington University in St. Louis, St. Louis, Missouri;; 3 Cyclotron Facility, School of Medicine, Washington University in St. Louis, St. Louis, Missouri;; 4 Department of Orthopaedics, Johns Hopkins University, Baltimore, Maryland;; 5 Research and Development Service, Baltimore VA Medical Center, Baltimore, Maryland;; 6 Department of Pharmacology, UCLA, Los Angeles, California;; 7 Department of Clinical Sciences, Lund University, Lund, Sweden;; 8 Department of Radiation Oncology, School of Medicine, Washington University in St. Louis, St. Louis, Missouri;; 9 Division of Gastroenterology and Hepatology, Department of Medicine, School of Medicine, Johns Hopkins University, Baltimore, Maryland;; 10 Department of Oncology, School of Medicine, Johns Hopkins University, Baltimore, Maryland;; 11 Center for Comparative Medicine, Baylor College of Medicine, Houston, Texas; and; 12 Department of Biomedical Engineering, Washington University in St. Louis, St. Louis, Missouri

**Keywords:** ^223^Ra, amiloride, ion channel, gastrointestinal, bone

## Abstract

[^223^Ra]RaCl_2_ is the first approved α-particle–emitting therapy and is indicated for treatment of bone metastatic castration-resistant prostate cancer. Approximately half the dose is absorbed into the gastrointestinal tract within minutes of administration, limiting disease-site uptake and contributing to toxicity. Here, we investigated the role of enteric ion channels and their modulation for improved therapeutic efficacy and reduced side effects. **Methods:** Using primary human duodenal organoids (enteroids) as in vitro models of the functional gastrointestinal epithelium, we found that amiloride (epithelial sodium ion channel blocker) and NS-1619 (K^+^ channel activator) presented significant effects in ^223^Ra membranal transport. Radioactive drug distribution was evaluated for lead combinations in vivo and in osteosarcoma and prostate cancer models. **Results:** Amiloride shifted ^223^Ra uptake in vivo from the gut and nearly doubled the uptake at sites of bone remodeling. Bone tumor growth inhibition with the combination as measured by bioluminescent imaging and radiography was significantly greater than that with single agents alone, and the combination resulted in no weight loss. **Conclusion:** This combination of approved agents may readily be implemented as a clinical approach to improve the outcomes of bone-metastatic cancer patients with the benefit of ameliorated tolerability.

## Visual Abstract

**Figure:**
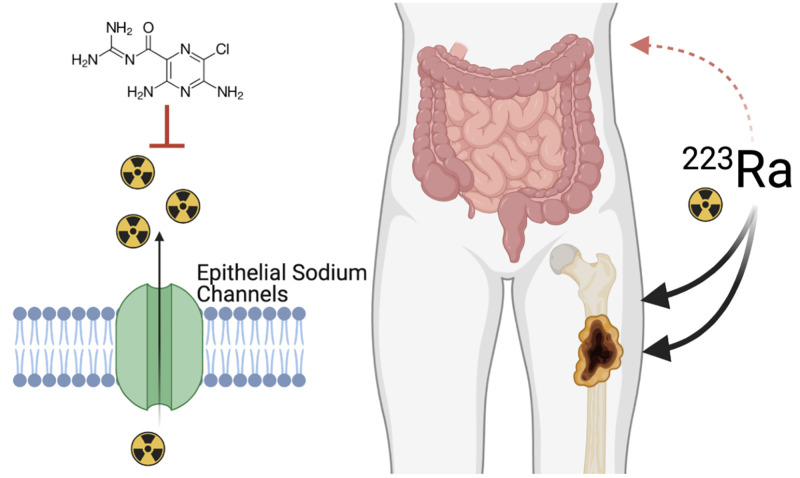

Prostate adenocarcinoma is the most common noncutaneous cancer diagnosed in men. Early treatment with radiation or surgery has a high rate of success; however, recurrent disease is incurable. Prostate cancer frequently metastasizes to the bone, where physical and microenvironmental factors make both focal and systemic treatments less effective. Bone metastases cause debilitating pain and fractures, displace the hematologic compartment, and ultimately cause death (*1*,*2*).

In 2013, [^223^Ra]RaCl_2_ became the first approved therapy specifically for improved survival in men with bone metastases from castration-resistant prostate cancer. This first-in-class α-particle–emitting therapy has a manageable hematologic profile, significantly delays symptomatic skeletal events, and extends overall survival in line with next-generation antiandrogens (*3*,*4*). α-particles deposit megaelectron volts of energy to cells adjacent to the site of decay. Localizing to sites of bone turnover apposite to skeletal metastases, ^223^Ra has a relative biological effect several fold more effective than that of conventional external-beam radiation, and treatment is not limited by factors such as hypoxia (*5*).

Despite the ablative potential of α-therapy, survival improvements are modest. This may be due in part to insufficient delivery of radioisotope, as well as dose limitations for its safe application. Radium is an alkaline earth metal that accumulates at sites of bone turnover; however, nearly half the administered activity accumulates within minutes in the gastrointestinal tract (*6*,*7*). This accumulation reduces the activity reaching sites of disease and is associated with radiotoxic sequelae in the gastrointestinal tract from ^223^Ra and daughters’ decay (*8*). Although gastrointestinal disposal is known (*9*,*10*), the biologic basis for transport of ^223^Ra from the blood to the lumen has not been investigated, and the overwhelming majority of metal-transport literature concerns apical-to-basolateral absorption.

Investigations of ^223^Ra clearance in small animals recapitulates clearance found in humans, with more than 50% of the initial activity cleared by 24 h (*11*–*13*). In this work, we evaluated radium ion transport to the gut after intravenous administration and the impact of pharmacophores on modulation of this flux. Using in vitro enteric systems and in vivo evaluation of organ distribution, we showed that gastrointestinal uptake can be inhibited by amiloride, a clinically approved epithelial sodium ion channel (ENac) blocker. When used in combination, ^223^Ra is rerouted from radiosensitive organs to sites of active bone turnover at metastases, providing therapeutic benefit in a prostate cancer model of bone metastasis and reduced treatment-induced toxicity. These data motivate clinical evaluation of this approach for improved α-therapy.

## MATERIALS AND METHODS

### Chemicals and Isotope Production

Chemicals were purchased from Sigma-Aldrich, unless otherwise noted. [^223^Ra]RaCl_2_ was produced using a previously described method (*14*) wherein pure ^223^Ra was generated from the parent isotope ^227^Th or ^227^Ac source provided by Oak Ridge National Laboratory, Department of Energy. Briefly, 370–740 kBq (10–20 μCi) of parent isotope were adsorbed on an anionic polymeric resin (1 × 8, Dowex) and eluted under mild acidic conditions (methanol:nitric acid [2N] 80:20) to isolate ^223^Ra-nitrates. The final sample was suspended in sodium citrate (0.03 M) and saline (150 mM) and assessed by a high-purity germanium detector, showing undetectable isotopic parent breakthrough and a ^223^Ra radiochemical purity of over 99%.

### In Vitro Studies

Cultured human duodenal enteroids and Caco-2/BBe1 monolayers were grown on Transwell (Corning) polymeric filters. This permitted quantification of ^223^Ra transcellular transport, mimicking the physiologic excretion of ^223^Ra. Monolayer growth conditions, integrity tests, and counting methodology are detailed in the supplemental materials (available at http://jnm.snmjournals.org) (*15*–*17*).

### In Vivo Studies

Animal experiments were conducted in accordance with Institutional Animal Care and Use Committee–approved protocols of the Johns Hopkins University School of Medicine and Washington University School of Medicine (*18*).

### Autoradiography

Aged male C57BL/6 mice (>35 wk) were retroorbitally administered a 250 kBq/kg dose of [^223^Ra]RaCl_2_ and killed at 10 min or at 1, 4, 24, or 48 h. The whole gastrointestinal tract from stomach to rectum was rapidly harvested and placed on cellophane (approximately 35-μm thickness) over autoradiographic phosphor screens for 90 min of exposure protected from light and read on a Cyclone Phosphor Imager (Packard) at 300 DPI (*19*).

### Immunohistologic Staining

Male C57BL/6 mice (Charles River) aged 20–30 wk were treated with GoLYTELY (24 g/350 mL; Braintree Laboratories) 2 h before intravenous administration of [^223^Ra]RaCl_2_ (250 kBq/kg). Portions (1 cm) of duodenum, jejunum, ileum, and colon were harvested at 24 h, embedded in optimal-cutting-temperature compound, and flash-frozen. Transversal sections were cut using a Leica 1860 cryomicrotome at 10 μm and then fixed by immersion in 10% paraformaldehyde (15 min). Stained slides (supplemental materials) were mounted using 4,6-diamidino-2-phenylindole or tetramethylrhodamine-phalloidin medium and imaged (Zeiss Axioplan M1 μmanager 2.0) (*20*).

### Radioactive Organ Distribution

Male C57BL/6 mice aged more than 12 wk (*n* = 6) were dosed intraperitoneally with amiloride (12.5 mg/kg—150 μL of a 2% v/v dimethyl sulfoxide solution) or NS-1619 (1 mg/kg—150 μL of a 2% v/v dimethyl sulfoxide solution) 1 h before retroorbital [^223^Ra]RaCl_2_ injection (3.7 kBq in 100 μL of citrate saline buffer). Tissues were harvested at 15 min and at 1, 4, 24, 48, and 240 h after administration of radionuclide, weighed, and γ-counted with an energy range of 150–350 keV, at 10 min per sample (Wizard2; Perkin Elmer). Standard samples (10% of injected activity) were measured, and percentage injected activity (%IA) normalized to tissue weight (%IA/g) was reported. Dosimetry was computed from these data, as detailed in the supplemental materials (*21*–*23*).

### Tumor Distribution, Response, and Toxicity Studies

Male Rag2-Il2rg double-knockout mice more than 12 wk old (R2G2; Envigo) were implanted with 5E6 Saos-2 osteosarcoma cells in the flank or with 2.5E5 luciferase-expressing C4-2B cells in the tibia (*24*). Animals were monitored by planar radiography (MX20; Hologic) and bioluminescence imaging (IVIS; Perkin Elmer) for xenograft progression and response. For uptake in the osteosarcoma model, mice were divided into 2 cohorts for [^223^Ra]RaCl_2_ injection alone or with amiloride. For C4-2B bone metastasis treatment, animals were randomized to receive amiloride or [^223^Ra]RaCl_2_ alone or together. For toxicologic studies, male FVB/NCr mice aged 14 wk (*n* = 5) were randomized into 4 cohorts treated with [^223^Ra]RaCl_2_ alone (3.7 kBq/100 μL), with amiloride alone (12.5 mg/kg intraperitoneally), with the combination of amiloride and [^223^Ra]RaCl_2_, or with saline (control). Weight changes and blood chemistry were monitored for 20 d, and histopathology was assessed by a certified veterinary pathologist. Treatment, imaging, and procedural details are found in the supplemental materials.

### Statistics

Significance was calculated with an unpaired 2-tailed *t* test with equal SD, using Prism Software (version 6D) by GraphPad. Significant differences were expressed at *P* values of less than 0.05.

## RESULTS

### Gastrointestinal Accumulation and Transit of ^223^Ra

**FIGURE 1.**
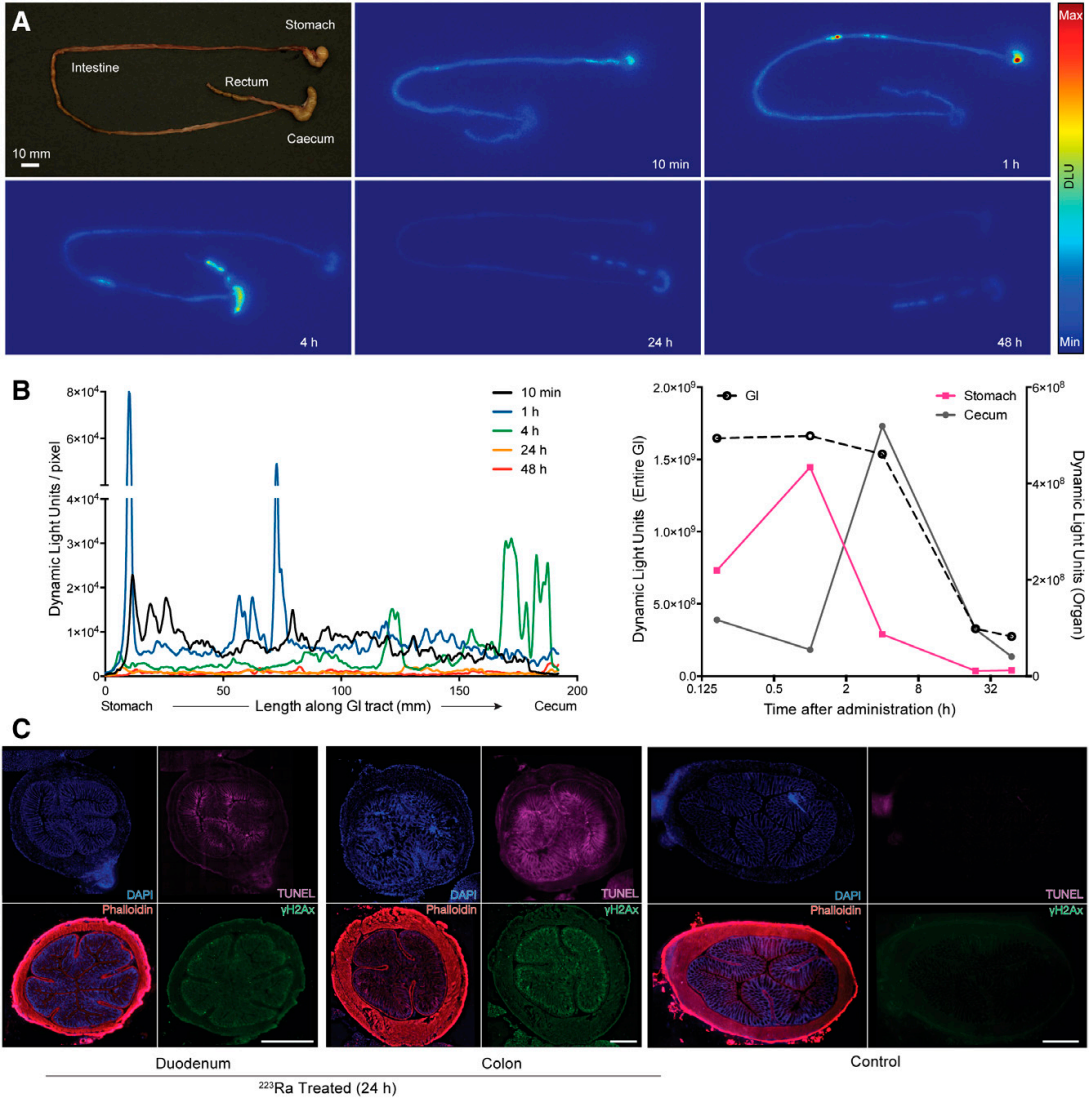
Gastrointestinal transit of [^223^Ra]RaCl_2_ and radiobiologic effects. (A) [^223^Ra]RaCl_2_ autoradiography of mouse gastrointestinal tract. (B) Signal intensity profiles from stomach to cecum displaying isotope migration (approximately 200 mm/animal) at indicated time points. Shown are upper (stomach) and lower (cecum) compartments and complete organ signal quantification, over time. (C) Immunofluorescence of duodenum and colon sections after treatment (left) and colonic section of saline control mouse (right). For each micrograph, stain and color are indicated: 4,6-diamidino-2-phenylindole (DAPI) for nuclei, phalloidin for cytoskeleton, terminal deoxynucleotidyl transferase 2′-deoxyuridine, 5′-triphosphate nick end labeling (TUNEL) marker for apoptosis, and γ-H2A histone family member X (γ-H2AX) for DNA damage. DLU = dynamic light units; GI = gastrointestinal.

It has been established that preclinical models accurately reflect the clinical distribution and kinetics of ^223^RaCl_2_ after intravenous administration (*11*–*13*,*24*,*25*). Most activity is transferred from circulation into the small intestine within minutes. To study the kinetics of elimination, we performed whole-organ autoradiography of the murine gastrointestinal tract at various times (10 min to 48 h) after administration of a 55 kBq/kg dose of [^223^Ra]RaCl_2_ (Fig. 1A).

Activity was detected at a high level as early as 10 min in the distal stomach and duodenum (Figs. 1A and 1B). Radium and daughters were then passed, for nearly complete clearance by 24 h. Immunofluorescence revealed radiobiologic damage generated by ^223^Ra transit (Fig. 1C). Tissue morphology was displayed using 4,6-diamidino-2-phenylindole and phalloidin, with DNA damage, cell apoptosis, and proliferation assessed using markers for γ-H2A histone family member X, terminal deoxynucleotidyl transferase 2′-deoxyuridine, 5′-triphosphate nick end labeling, and Ki-67 at 4 and 24 h (Supplemental Fig. 1). Radiation-related damage was identified in the duodenum and colon at 24 h, correlating with clearance kinetics.

### In Vitro Assessment of Active ^223^Ra Transport

**FIGURE 2.**
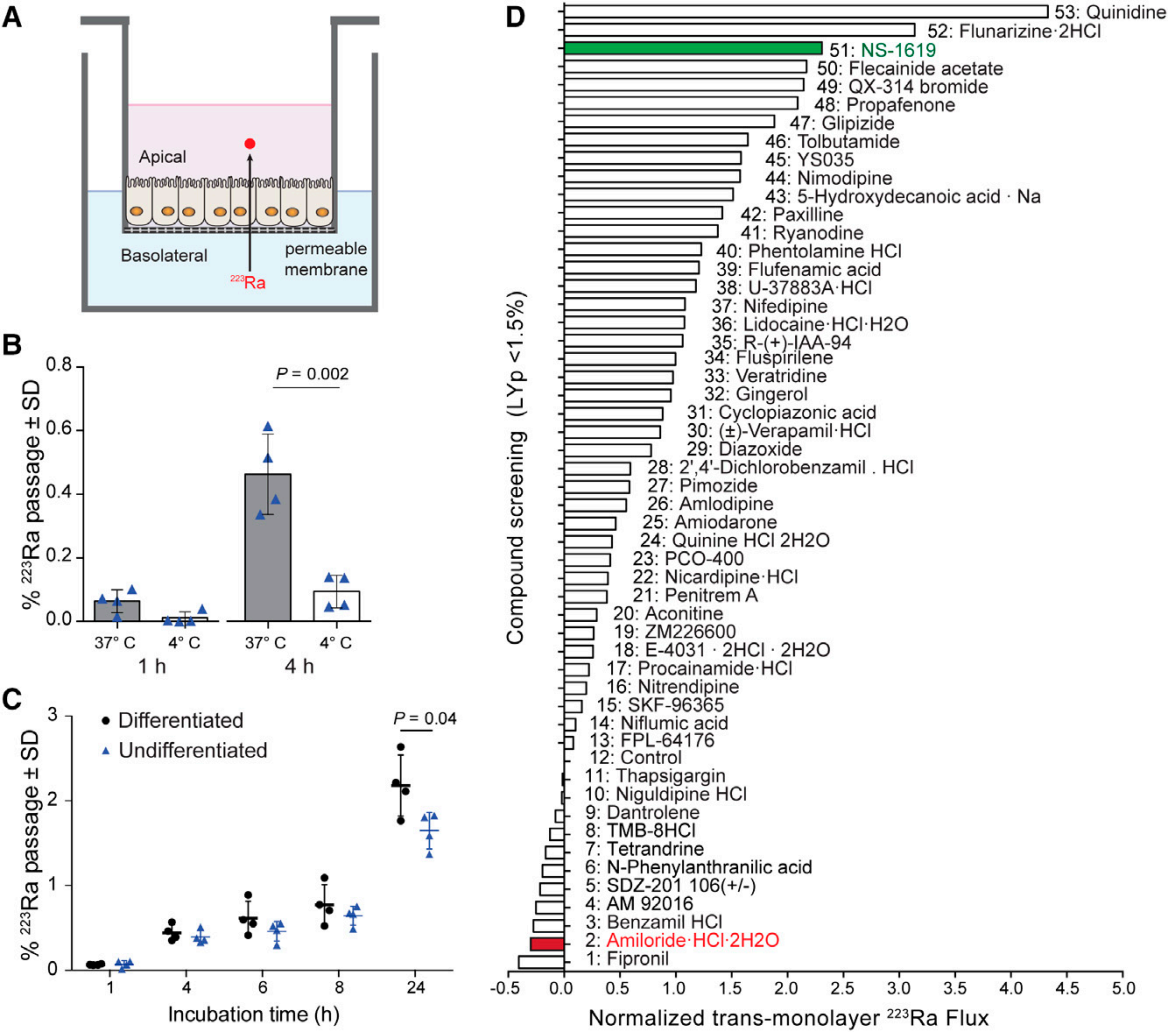
Active ^223^Ra transport through human gastrointestinal organoids. (A) Schematic representation of enteroid monolayers grown on permeable Transwell. (B) ^223^Ra passage to apical compartment normalized to initial dose in basolateral media, revealing temperature-dependent mechanism, with undifferentiated cryptlike enteroids. (C) Increased ^223^Ra transport measured using differentiated over undifferentiated enteroids as function of time (*P* < 0.05). (D) ^223^Ra flux normalized to untreated well as measured through caco-2 monolayers coincubated with library of 52 ion-channel inhibitors or activators. Caco-2 monolayer integrity was confirmed by pre- and postradioactive incubation measuring transepithelial/transendothelial electrical resistance and Lucifer yellow (Lucifer Yellow Permeability < 1.5%) readings. Average differential of radioactive counts (*n* = 3) has been normalized to ^223^Ra flux baseline exempt from treatment (no. 12 control well). Amiloride (red) and NS-1619 (green) proceeded for in vivo validation.

Transport from the basolateral to apical (luminal) compartment across human primary duodenal enteroid monolayers was used to model the blood and intestinal compartments (Fig. 2A). We first tested whether ^223^Ra transport was an active or passive process at physiologic and reduced temperatures (Fig. 2B). As expected, flux was suppressed at a lower temperature, in agreement with an active mechanism.

The location and maturation stage of enterocytes present characteristic ion channel profiles (*16*). Using undifferentiated (immature cryptlike enterocytes) and differentiated (mature villilike brush border enterocytes) enteroids grown from patient biopsy samples, we observed a significant preference for ^223^Ra transport across differentiated monolayers (2.2% ± 0.3%) over undifferentiated (1.6% ± 0.2%) (*P* < 0.05; Fig. 2C). The active and cell-type specific ^223^Ra transport led us to evaluate modulators to influence this flux.

Caco-2 cells in a μ-Transwell system were screened with 52 ion channel modulators incubated with [^223^Ra]RaCl_2_. Monolayer integrity was ensured by resistance and control-fluorescent compound measures. Forty molecules increased flux as compared with control, and 11 drugs inhibited transfer. The K^+^ channel activator NS-1619 was among the most effective in increasing ^223^Ra transport up to 3.3 ± 1.6-fold (*26*). In contrast, amiloride, an approved diuretic Na^+^/H^+^ channel blocker, decreased ^223^Ra transfer to 0.70 ± 0.14-fold. Despite electrochemical similarities, calcium channel blockers were ineffective ^223^Ra inhibitors.

### Modulation of ^223^Ra Distribution

**FIGURE 3.**
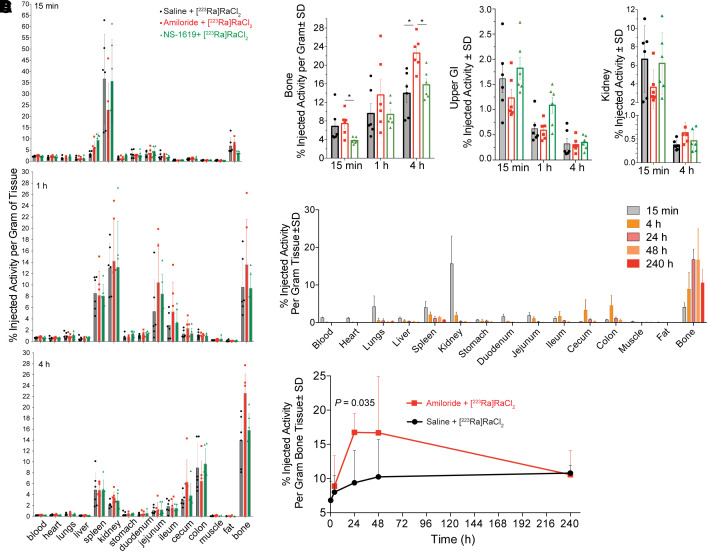
Evaluation of selected ion channel modulators with [^223^Ra]RaCl_2_. (A) Radioactive organ distribution of healthy male C57BL/6 mice randomized in 3 cohorts (*n* = 6) given amiloride before [^223^Ra]RaCl_2_, NS-1619 before [^223^Ra]RaCl_2_, and saline before [^223^Ra]RaCl_2_. Several organs displayed significant differences in ^223^Ra uptake, including 1.5-fold higher bone localization with amiloride. Differences in scale can be seen for 15-min data (values reported in supplemental materials). (B) ^223^Ra bone activity uptake (%IA/g) at 15 min for NS-1619 combination is half that of control and amiloride-treated groups. In contrast, amiloride-treated group shows significantly higher bone uptake than does control group (*P < 0.05). Upper gastrointestinal radioactive uptake reflects higher content for NS-1619–treated animals at 15 min (*P < 0.05). Kidney uptake across cohorts at 15-min time point shows decrease for amiloride group. (C and D) Whole-organ distribution (C) and bone focus over 10-d time course (D) comparing amiloride combination to [^223^Ra]RaCl_2_; activity uptake at 24 h significantly differs between the 2 groups (*P* = 0.035).

The pharmacokinetic impact on ^223^Ra by lead compounds to either promote (1 mg/kg dose of NS-1619) or inhibit (12.5 mg/kg dose of amiloride) enteric transit was assessed by biodistribution. Ion channel modulation is transient (*27*,*28*), and an initial dose and schedule-finding study was performed (supplemental materials and Supplemental Figs. 2 and 3). Organ activity levels from skeletally mature animals are shown as %IA/g (Fig. 3A; Supplemental Table 1). Increasing amounts of activity in the bone after radiopharmaceutical administration were accompanied by kidney and upper gastrointestinal uptake (stomach, duodenum, and jejunum), which was passed (Fig. 1A).

Intestinal absorption was significantly greater at early time points: stomach uptake at 15 min with NS-1619 (2.73 ± 0.44 %IA/g) was 2-fold over that in control animals (1.39% ± 0.6%), with a concomitant decrease in osseous uptake (*P* < 0.05; Figs. 3A and 3B). In contrast, the amiloride combination decreased gastrointestinal and renal uptake of ^223^Ra and delayed kinetics of transport through the gastrointestinal tract. Upper gastrointestinal uptake was lower than that of the control and ion channel activator combination at 15 min (%IA), with greater ^223^Ra in the tibia, reaching 22.62 ± 3.62 over that of the control level of 13.96 ± 4.32 %IA/g (*P* < 0.05).

We evaluated changes beyond the acute setting out to the physical half-life of the radionuclide (Figs. 3C and 3D; Supplemental Table 2). Bone uptake for the combination with amiloride, at 18.9 ± 8.3 %IA/g, was nearly twice that of the control group, at 10.2 ± 5.4 %IA/g at 48 h. Dynamic turnover reduced activity to a nearly equal 10 %IA/g at 10 d (Supplemental Fig. 4). Estimating absorbed doses on the organ scale, we measured a significant increase in therapeutic energy deposition at the intended osseous sites, 1.43 Gy, compared with 1.03 Gy for [^223^Ra]RaCl_2_-only treatment (supplemental materials).

### Bone Tumor Targeting and Therapeutic Outcomes

**FIGURE 4.**
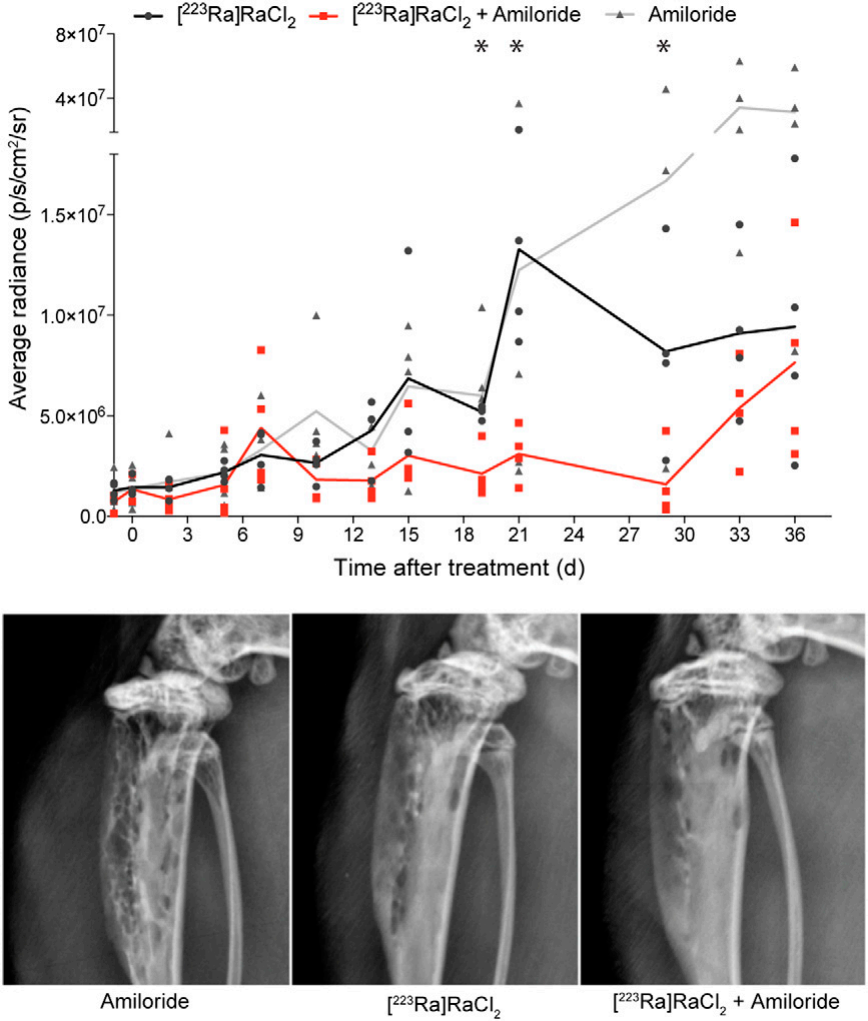
Tumor growth inhibition and monitoring of C4-2B bone-inoculated animals treated with amiloride, combination of amiloride plus [^223^Ra]RaCl_2_, and [^223^Ra]RaCl_2_ alone. Average bioluminescent radiance measured using luciferase-expressing C4-2B cells inoculated in tibia shaft shows superior tumor growth inhibition for combination cohort. *P < 0.05 at days 19, 21, and 29 comparing [^223^Ra]RaCl_2_ with combination. Animals on combination lost less weight and regained that mass faster than with either agent alone (Supplemental Fig. 6). Representative radiograph of tibia 35 d after injection demonstrating degradation for control amiloride as compared with radiotherapy and combination cohorts.

Quantification of radionuclide localization in rodent models of bone metastasis is complicated by continued normal bone turnover, as the epiphyseal plates of mice do not fuse (*13*). To address this issue, we tested heterotopic ossification using an osteosarcoma model. Mineralized subcutaneous tumors were identified using radiography and ^18^F-NaF PET (Fig. 4). Tissues of interest were excised, counted, and weighed at 24 h after [^223^Ra]RaCl_2_ (55 kBq/kg) with or without combination amiloride (12.5 mg/kg). At that time point, no difference in kidney or gastrointestinal uptake was noted; however, tibia (*P* < 0.005) and osteosarcoma (*P* < 0.01) samples from the combination treatment had increased ^223^Ra accumulation (Supplemental Fig. 5).

An intratibial inoculation of luciferase-expressing castration-resistant C4-2B cells (*29*) was used to test impact in the therapeutic setting by bioluminescence imaging and radiography (Supplemental Fig. 6). Animals were randomized to receive [^223^Ra]RaCl_2_ alone, [^223^Ra]RaCl_2_ in combination with amiloride, or amiloride alone. An approximately 30-fold increase in radiance was observed for the amiloride-only group at 33 d after treatment, indicating no antitumor effect for the mixed osteolytic/blastic C4-2B lesions (Fig. 4; Supplemental Fig. 6). Body mass was monitored and showed that the combination resulted in less weight loss and a more rapid regain rate. These data show that both a disease burden reduction and sparing of the gastrointestinal tract from the transient initial dose enables faster recovery. At termination, radiographs displayed reduced osteolysis due to combination treatment.

### Toxicologic Evaluation of Combination

**FIGURE 5.**
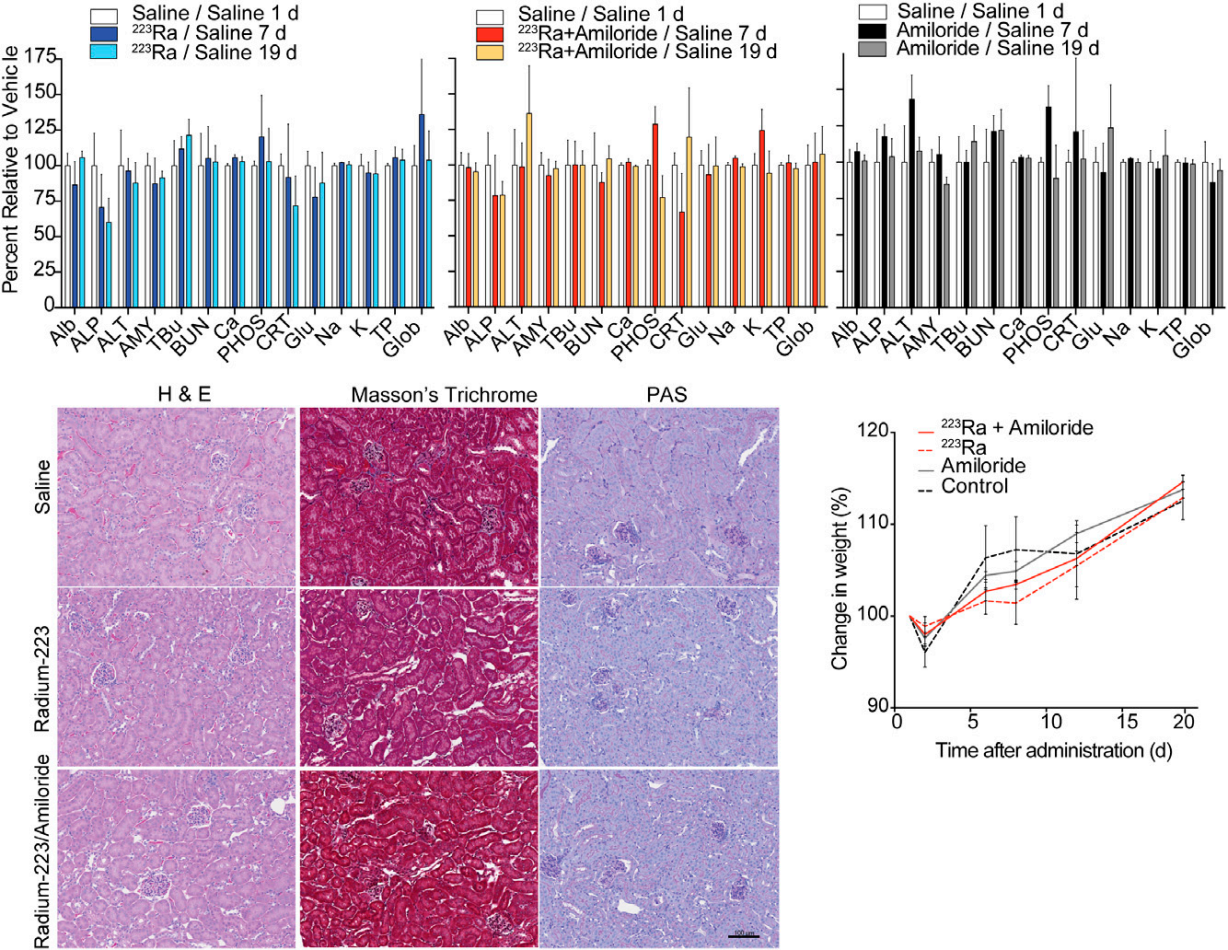
Toxicologic effects of single or combination treatment: blood chemistry markers for control saline, [^223^Ra]RaCl_2_ or amiloride alone, and combination at 1, 7, and 19 d after administration. For [^223^Ra]RaCl_2_ and combined therapy, amiloride, ALP, and creatinine present significant drop at 7 d as compared with control saline cohort, with no other noticeable differences found. Kidney pathology by hematoxylin and eosin (H & E), Masson trichrome, and periodic acid–Schiff (PAS) staining at 20 d after treatment indicates no morphologic differences. Weight monitoring demonstrated indistinguishable gain for all groups. ALP units are U/L. ALb = albumin (g/L); ALT = alanine amino transferase (U/L); AMY = amylase (U/L); TBu = total bilirubin (μmol/L); BUN = blood urea nitrogen (mmol/L); Ca = calcium (mmol/L); PHOS = phosphate (mmol/L); CRT = total protein (g/L); Glob = globulin (g/L).

To investigate potential combination-induced sequelae, we randomized mice into 4 cohorts to receive saline, amiloride, [^223^Ra]RaCl_2_, or the combination. Clinical chemistry analysis was conducted using a multiparameter blood panel over 19 d. Postmortem kidney tissues were histopathologically examined to identify potential treatment-related damage, and weight was monitored throughout (Fig. 5). Most clinical chemistry values across all cohorts did not deviate significantly from those of the vehicle cohort. Levels of alkaline phosphatase (ALP) and creatinine were decreased in both radium-treatment groups at day 7 (creatinine, 11–14 μmol/L; ALP, 47–51 u/L), as compared with the vehicle group (creatinine, 16 μmol/L; ALP, 66.7 u/L). Creatinine recovered to control levels after 7 d, whereas ALP remained suppressed. This finding may reflect clinical experience with patients on [^223^Ra]RaCl_2_ (*30*,*31*).

The slight increase in renal excretion of ^223^Ra measured from the combination at 4 h after injection (Figs. 3A and 3B) led us to investigate kidney status by standard histopathologic examination. Morphologic analysis of tissue sections by hematoxylin and eosin and periodic acid–Schiff staining, as well as renal fibrosis by Masson trichrome staining, indicated no aberrant pathology across groups at 20 d. Additionally, all animals continued increased weight for all groups (Fig. 5C). Clinical chemistries and tissue assessments confirm no evident toxicity incurred by the combination.

## DISCUSSION

After decades of development in the field of targeted nuclear therapy, [^223^Ra]RaCl_2_ became the first α-therapy to be approved by the European Medicines Agency and the Food and Drug Administration, in 2013 (*32*). This treatment improves survival, reduces fracture incidence, and decreases bone pain in men with prostate cancer metastatic to bone. Although the treatment is well tolerated, responses are moderate and ^223^Ra and its daughters’ sequestration in the gastrointestinal tract can result in treatment cessation (*8*) and reduces the dose to disease sites.

Radium research has focused on bone and metastases (*11*,*24*,*33*–*35*). As a starting point to assess the distribution of [^223^Ra]RaCl_2_, we validated preclinical models to approximate human clearance (*6*). Our work in skeletally mature mice recapitulated ^223^Ra distribution in humans (*11*–*13*), and whole-tract imaging of excised gastrointestinal showed pharmacokinetics similar to that of patients, with uptake in the stomach and duodenum as early as 10 min after injection (Fig. 1). Immunofluorescence revealed radiation damage at the cellular level associated with apoptosis and DNA damage activity. Specifically, the γ-H2A histone family member X signal of the duodenum and colon localized at the apical surface of the lumen, matching our visualization of the clearance.

The kinetics of this phenomenon led us to hypothesize that active cellular processes mediated by ion transport are involved and, further, that pharmacologic modulation may decrease toxicity and improve therapeutic outcomes. Radium is an alkaline earth metal, yet its transport was not altered by Ca^2+^ channel blockers. Verapamil, a prototypical calcium channel inhibitor, had little effect in vitro or in vivo (Supplemental Fig. 7). Instead, Na^+^/H^+^ channel modulators such as amiloride and benzamil had potent inhibitory effects and did not disturb enteroid integrity. Fipronil showed even greater efficacy in ^223^Ra blockade; however, this insecticide was excluded from further study. Diverse families of ion channels are expressed throughout the gastrointestinal tract, regulating critical functions of nutrient and fluid secretion and absorption and of motility. The modulators tested affect different families of ion channels and do not confirm an exclusive ^223^Ra-dependent transporter or class, and identification of specific ^223^Ra-transport channels requires further investigation.

In vivo data on lead compounds confirmed the cellular assay results (Fig. 3). Study parameters were determined from a dose-and-timing study demonstrating that intraperitoneal administration of amiloride 1 h before [^223^Ra]RaCl_2_, at a dose of 12.5 mg/kg, robustly inhibited intestinal transport and increased bone accumulation (Supplemental Figs. 3 and 4). Further optimization for other combinations may be required. Amiloride combined with [^223^Ra]RaCl_2_ transiently decreased gastrointestinal accumulation and drove a substantial increase to bone (nearly 2-fold). In contrast, NS-1619 increased acute upper gastrointestinal sequestration. Increased uptake in the skeleton with the radium and amiloride combination results in a greater total energy deposited (Supplemental Table 3).

These results encouraged us to pursue study of amiloride with [^223^Ra]RaCl_2_ in the disease setting after first confirming the osteoid targeting properties of the combination in an osteosarcoma model of pathologic bone. A significantly decreased bone tumor burden in the ^223^Ra cohorts was observed, with a more rapid and deeper response in the combination cohort. These outcomes, after a single administration, were accompanied by faster renormalization of weight, and we presume recovery is due to decreased tumor burden and mitigated gastrointestinal damage. Clinical chemistry and histopathologic analysis were also performed, showing that creatinine values followed the trend of regained weight. Amiloride side effects include dizziness, muscle spasms, and nausea, and patients with impaired renal function will need to be closely monitored.

These results are impactful for radium patients, as many men bear substantial disease, have faced several prior lines of potentially toxic cancer therapy, and have comorbidities engendering careful patient management. We anticipate that the increased tolerability and dose accumulation at sites of disease are likely to synergize over the course of the 6 cycles to extend both treatment duration and magnitude of response.

## CONCLUSION

Investigations using cellular systems and in vivo models demonstrated that ^223^Ra uptake is an active cellular process. The novel combination of amiloride with [^223^Ra]RaCl_2_ improved the efficacy of α-therapy. The attendant reduction in absorbed dose to other sites ameliorated tolerability issues and was found to minimally impact at-risk organs and clinical chemistry profiles. This combination of 2 approved agents warrants further evaluation in the patient management setting.

## DISCLOSURE

This work was supported by the SNMMI Junior Faculty Fund, 2015 (Diane Abou); the Patrick C. Walsh Prostate Cancer Research Fund (Daniel Thorek); and the National Cancer Institute (R01CA229893, R01CA201035, and R01CA240711 [Daniel Thorek]). The Integrated Physiology Core of the Hopkins Conte Digestive Disease Basic and Translational Research Core Center (P30DK-089502; Nicholas Zachos) provided human enteroids and media. No other potential conflict of interest relevant to this article was reported.

## Notes

KEY POINTS **QUESTION:** Is it possible to inhibit the rapid uptake of ^223^Ra in the gastrointestinal tract in order to drive a greater dose to bone metastases? **PERTINENT FINDINGS:** Amiloride combined with [^223^Ra]RaCl_2_ decreased gastrointestinal accumulation and resulted in a substantial increase (nearly 2-fold) in bone uptake. The combination improved the efficacy of therapy to delay metastatic growth while presenting no apparent toxicity. **IMPLICATION FOR PATIENT CARE:** This novel strategy has significant potential to improve the on-target ^223^Ra effect while reducing treatment-induced toxicities and weight loss.
